# Resting State Functional Connectivity MRI among Spectral MEG Current Sources in Children on the Autism Spectrum

**DOI:** 10.3389/fnins.2016.00258

**Published:** 2016-06-09

**Authors:** Michael Datko, Robert Gougelet, Ming-Xiong Huang, Jaime A. Pineda

**Affiliations:** ^1^Cognitive Science, University of California San DiegoLa Jolla, CA, USA; ^2^Neurosciences, University of California San DiegoLa Jolla, CA, USA; ^3^Department of Radiology, University of California San DiegoLa Jolla, CA, USA

**Keywords:** autism, magnetoencephalography, fMRI, functional connectivity, multimodal

## Abstract

Social and communicative impairments are among the core symptoms of autism spectrum disorders (ASD), and a great deal of evidence supports the notion that these impairments are associated with aberrant functioning and connectivity of various cortical networks. The present study explored the links between sources of MEG amplitude in various frequency bands and functional connectivity MRI in the resting state. The goal of combining these modalities was to use sources of neural oscillatory activity, measured with MEG, as functionally relevant seed regions for a more traditional pairwise fMRI connectivity analysis. We performed a seed-based connectivity analysis on resting state fMRI data, using seed regions derived from frequency-specific amplitude sources in resting state MEG data in the same nine subjects with ASD (10–17 years of age). We then compared fMRI connectivity among these MEG-source-derived regions between participants with autism and typically developing, age-matched controls. We used a source modeling technique designed for MEG data to detect significant amplitude sources in six frequency bands: delta (2–4 Hz), theta (4–8 Hz), alpha (8–12 Hz), beta (12–30 Hz), low gamma (30–60 Hz), and high gamma (60–120 Hz). MEG-derived source maps for each participant were co-registered in standard MNI space, and group-level source maps were obtained for each frequency. For each frequency band, the 10 largest clusters resulting from these *t*-tests were used as regions of interest (ROIs) for the fMRI functional connectivity analysis. Pairwise BOLD signal correlations were obtained between each pair of these ROIs for each frequency band. Each pairwise correlation was compared between the ASD and TD groups using *t*-tests. We also constrained these pairwise correlations to known network structures, resulting in a follow-up set of correlation matrices specific to each network we considered. Frequency-specific MEG sources had distinct patterns of fMRI resting state functional connectivity in the ASD group, but perhaps the most significant was a finding of hypoconnectivity between many sources of low and high gamma activity. These novel findings suggest that in ASD there are differences in functionally defined networks as shown in previous fMRI studies, as well as between sets of regions defined by magnetoencephalographic neural oscillatory activity.

## Introduction

Autism spectrum disorders (ASD) are characterized by social and communicative impairments, as well as repetitive and stereotyped behaviors (DSM-V, American Psychiatric Association (APA), 2013). It is well established that these impairments may result from aberrant anatomy and functional connectivity, defined as inter-regional correlations in the time-course of the fMRI BOLD signal (Biswal et al., 1995), within and between various cortical networks (Vissers et al., 2012). These atypical patterns of functional connectivity may underlie the disordered information integration characteristic of the ASD brain (Brock et al., 2002), therefore accounting for the myriad symptoms along the autism spectrum (Belmonte et al., 2004).

Unsurprisingly, functional connectivity in the ASD population is largely idiosyncratic across tasks (Vissers et al., 2012), methodologies (Müller et al., 2011), and behavioral symptoms (Hahamy et al., 2015). Findings include hypoconnectivity in some systems (Just et al., 2004, 2007; Kana et al., 2006, 2007), and hyperconnectivity in others (Welchew et al., 2005; Mizuno et al., 2006; Turner et al., 2006; Noonan et al., 2009; Shih et al., 2010). More recent studies have refined the characterization of functional connectivity in ASD, highlighting the contrast between within-network and out-of-network connectivity patterns, and argue for reduced within-network integration along with increased out-of-network connectivity, which ultimately results in reduced network segregation in ASD (Fair et al., 2009; Rudie et al., 2011, 2013; Shih et al., 2011; Fishman et al., 2014; Nebel et al., 2014). This characterization is supported by observations from Keown et al. (2013) and Supekar et al. (2013), who found functional hyperconnectivity in children with ASD across multiple networks, and from Pérez Velázquez and Galán (2013), who found an increase in information gain in the absence of external stimuli in ASD subjects, possibly as a consequence of hyperconnectivity and network cross-talk.

Current understanding about the neurophysiological etiology of ASD may shed light on the functional connectivity abnormalities observed during rest and task performance. It has been hypothesized that in ASD, cortical GABAergic interneurons may fail to preserve proper excitation/inhibition dynamics during development, causing irregularities in synaptic pruning and network maturation (Hensch, 2005; Coghlan et al., 2012; Rosenberg et al., 2015). In a recent study on adults with ASD, low GABA concentrations in visual cortex were shown to correlate with decreased performance on a binocular rivalry task (Robertson et al., 2016). In addition to these GABAergic interneuron dysfunctions, abnormalities in cortical minicolumns in the frontal cortex (Casanova et al., 2002), as well as enlarged frontal gray and white matter (Carper and Courchesne, 2005; Courchesne and Pierce, 2005), may also contribute to the functional connectivity issues in ASD. Whatever the sources of the abnormalities seen in ASD are, functional connectivity remains an important metric in elucidating the neurophysiological substrates of the disorder.

Non-invasive electrophysiological measures like EEG and MEG have been crucial in providing converging evidence for functional connectivity abnormalities in ASD (Vissers et al., 2012). These biophysical signals are often examined in spectral bands prescribed in the literature: delta (0–4 Hz), theta (4–8 Hz), alpha (8–12 Hz), beta (12–30 Hz), low gamma (30–60 Hz), and high gamma (60+ Hz), many of which exhibit abnormal patterns in ASD compared to typically developing controls. Subjects on the spectrum show reduced interhemispheric coherence in the gamma band (Peiker et al., 2015), a finding that provides support for the weak central coherence hypothesis of autism. Barttfeld et al. (2011), on the other hand, found distinct EEG connectivity patterns within the delta range during rest in an ASD population. These subjects lacked long-range connections, with most prominent deficits in fronto-occipital networks, and increased short-range connections in lateral-frontal networks. But while electrophysiological approaches provide important results like these on their own, the complementary use of MEG and fMRI can provide both millisecond and millimeter precision capable of spanning the multiple orders of temporal and spatial magnitudes involved in neocortical processing (Dale et al., 2000; Dale and Halgren, 2001; Liu et al., 2006; Salmelin and Baillet, 2009).

Research on these modalities suggests that the cortical neuronal activity that generates measurable electromagnetic fields imposes metabolic demands that are discernable by fMRI BOLD (Dale et al., 2000; Dale and Halgren, 2001; Logothetis et al., 2001; Arthurs and Boniface, 2002; Logothetis, 2002, 2003, 2008; Logothetis and Wandell, 2004). In particular, power in the mid-gamma band (60–120 Hz) has been shown to positively correlate with BOLD signals, whereas beta (13–30 Hz) power shows a negative correlation with BOLD (Conner et al., 2011). The higher co-localization of gamma band synchronous activity with fMRI BOLD becomes relevant in the discussion of ASD when considering that GABAergic interneurons are responsible for generating the gamma cortical oscillation (Cardin et al., 2009), and there may be dysfunction among GABAergic interneurons in ASD (Coghlan et al., 2012). Indeed, the combined use of MEG and fMRI to investigate functional connectivity could provide important new insights into the functional properties of gamma activity in ASD. Yet despite the complementarity of these two methods, there is a lack of cross-modal investigations that link measures of MEG and fMRI connectivity in autism.

The present study explored the links between MEG current source amplitudes in various frequency bands and functional connectivity MRI (fcMRI) in a resting state. More specifically, we performed a seed-based connectivity analysis on fMRI data, with seed regions based on amplitude sources from MEG data within a subgroup of subjects. We then compared connectivity among these regions between participants diagnosed with ASD and typically developing controls. First, we predicted that MEG amplitude sources for each frequency band would be located in areas previously associated with those frequencies for both ASD and typically developing controls. For instance, we predicted that alpha sources would be concentrated in visual areas, while areas associated with the mu rhythm centered more on somatosensory and premotor cortex (Pfurtscheller et al., 2006; Bernier et al., 2007). Furthermore, beta sources were expected near the central gyrus but also to extend to more frontal areas (Jensen et al., 2005). We predicted theta sources would be located in midline frontal and prefrontal areas (Iramina et al., 1996), while gamma sources were expected to show a sparse and widely distributed pattern (Cardin et al., 2009). We further predicted that ASD would show abnormal resting state fMRI connectivity patterns among regions corresponding to MEG current sources in the various frequency bands. Based on previous observations of reduced within-network integration (Rudie et al., 2011, 2013), we predicted that MEG current sources falling within the same networks would show hypoconnectivity in ASD.

## Methods

### Participants

Nine participants with high-functioning autism (mean age = 13.1 ± 2.59, range = 10–17; 1 female; mean WASI IQ = 96.1 ± 15.6, range = 72–121; mean ADOS Communication and Social Interaction score = 13.7 ± 3.14, range = 12–20) were scanned with both MEG and fMRI. A clinical psychologist collaborator verified autism diagnoses through the administration of the ADOS test (Rutter et al., 2012). Nine age-matched, typically developing (TD) participants (Mean age = 10.6 ± 2.75, range = 8–16; 3 female; mean WASI IQ = 118.33 ± 13.1, range = 103–138) completed both resting state fMRI and anatomical MRI scans, but not MEG scans. TD participants had no major diagnoses and did not have ASD siblings or parents. Groups did not differ significantly for age (*p* = 0.14), but were not matched for WASI IQ (*p* = 0.005). All participants gave informed consent or assent, and read forms describing the nature of the experiment and their rights as participants. There were two different age-appropriate forms, one for children ages 7–12 and the other for ages 13–17.

### MEG data collection and preprocessing

Participants in the ASD group completed two back-to-back, 4-min resting state MEG scans, during which they were instructed to keep their eyes open with their gaze directed at a fixation point, and let their mind wander. MEG data were collected for ASD participants using the Elekta/Neuromag Vectorview whole-head MEG system. Data were sampled at 1000 Hz and were bandpass filtered between 0.1 and 330 Hz. Eye blinks and eye movements as measured via electrooculography (EOG), and heart activity as measured by electrocardiogram (ECG) were collected MEG sensor data were filtered for movement-related artifacts using the program MaxFilter (Taulu et al., 2005). Feeding continuous magnetic sinusoidal signals to five head position indicator coils allowed for subjects' head positions to be continuously collected. These signals are used to continuously adjust the coordinate transformation from the device to the head frame of reference, necessary for applying MaxFilter. These signals were removed from the data *post-hoc*, in addition to interference from other magnetic sources (e.g., 60 Hz line frequency and its harmonics). The spatiotemporal signal space separation (tSSS) method was used in this case to remove noise and artifacts originating from outside the brain (Taulu et al., 2004b, 2005). By continuously tracking the subjects' head positions, we minimized the influence of movement artifacts on our analyses, a common consideration when working with the pediatric ASD population.

Precautions were taken to ensure head stability: foam wedges were inserted between the subject's head and the inside of the unit. During collection, the head positions were measured to ensure that head movement across different sessions was < 5 mm (usually 2–3 mm). The ECG artifacts in the MEG data were also removed when the MEG data were passed through MaxFilter. This feature of MaxFilter has been described previously (Taulu et al., 2004a,b; Song et al., 2008).

Sensor data were co-registered with subjects' anatomical MRI scans for accurate source localization. To co-register the MEG with MRI coordinate systems, three anatomical landmarks (i.e., left and right preauricular points, and nasion) were measured for each subject using the Probe Position Identification system (Polhemus, USA). By identifying the same three points on the subject's MR images using MRILAB software developed by Elekta/Neuromag, a transformation matrix involving both rotation and translation between the MEG and MRI coordinate systems was generated. To increase the reliability of the MEG-MRI co-registration, ~300 points on the scalp were digitized with the Polhemus system, in addition to the three landmarks, and those points were co-registered onto the scalp surface of the MR images.

### MEG analysis

We used a source modeling technique (Fast-VESTAL) designed for MEG data, which consists of two steps (Huang et al., 2014). First, L1-minimum-norm MEG source images were obtained for the dominant spatial (i.e., eigen-) modes of the sensor-waveform covariance matrix. Next, accurate source time-courses were obtained using an inverse operator constructed from the spatial source images of Step 1. This approach has been successfully used to obtain comprehensive MEG source-magnitude images covering the entire brain for different frequency bands of resting-state brain rhythms (Huang et al., 2014). The six different frequency bands of interest in the present study were: delta (2–4 Hz), theta (4–8 Hz), alpha (8–12 Hz), beta (12–30 Hz), low gamma (30–60 Hz), and high gamma (60–120 Hz).

In the present study, each of the artifact-free, 8-min long, resting-state MEG sensor-space scans were bandpass filtered for each frequency band of interest. The sensor-waveform covariance matrix was calculated and used to obtain MEG frequency band source magnitude images that cover the whole brain for each subject following the fast-VESTAL procedure (Huang et al., 2014). An Objective Prewhitening Method was applied to remove correlated environmental noise and to select the dominant eigen-modes of the sensor-waveform covariance matrix (Huang et al., 2014).

**Figure 1:**
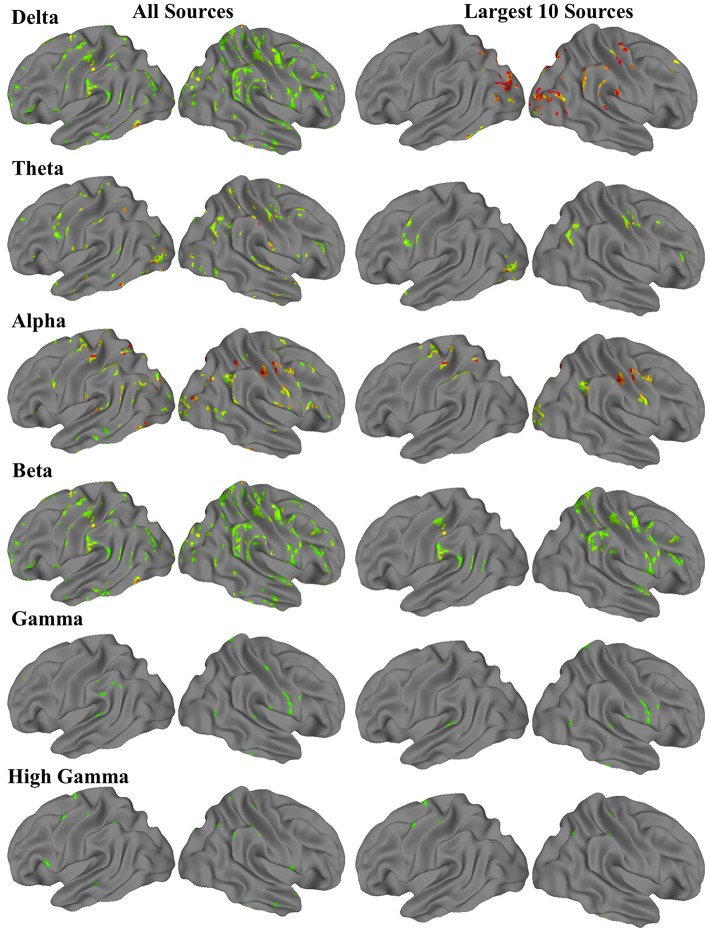
**Group-level MEG-derived source maps for each frequency band**. The first column shows all sources that were significant at corrected *p* < 0.01, while the second column shows the 10 sources with the most voxels.

For each frequency band, a three-dimensional image volume showing the locations and intensities of each amplitude source was obtained for each participant. These individual subject volumes were then aligned to standard Montreal Neurological Institute (MNI) space. Using the neuroimaging software suite Analysis of Functional Neuroimages (AFNI; Cox, 1996), we then performed a one-sample *t*-test with a whole-brain field-of-view to determine the most significant amplitude sources at the group level. The resulting images met an uncorrected voxel wise statistical threshold of *p* < 0.02, and were cluster corrected for multiple comparisons at *p* < 0.01. For each frequency band, the 10 clusters with the largest volumes resulting from these *t*-tests were saved as regions of interest (ROIs) for the fMRI functional connectivity analysis (Figure 1).

### fMRI data collection and preprocessing

For ASD subjects, resting state and anatomical imaging data were acquired on a GE 1.5T Excite MRI scanner. The anatomical scan was acquired as a standard high-resolution anatomical volume with a resolution of 0.94 × 0.94 × 1.2 mm^3^ using a T1-weighted 3D-IR-FSPGR pulse sequence. Functional T2^*^-weighted images were acquired with a single-shot gradient-recalled, echo-planar pulse sequence, as a single 7:48-min scan with 156 whole-brain volumes (TR = 3000 ms, TE = 40 ms, flip angle = 90°, FOV = 240 mm, 40 axial slices, 4 × 4 × 4 mm^3^ resolution).

For TD subjects, resting state and anatomical imaging data were acquired on a GE 3T MR750 scanner with an eight-channel head coil. High-resolution anatomical images were obtained using a standard T1-weighted inversion recovery spoiled gradient echo sequence (TR = 8.108 ms, TE = 3.172 ms, flip angle 8°, 172 slices, 1 mm^3^ resolution). Functional T2^*^-weighted images were acquired using a single-shot gradient-recalled, echo-planar pulse sequence, in one 6:10-min resting state scan consisting of 185 whole brain volumes (TR = 2000 ms, TE = 30 ms, flip angle 90°, FOV = 220 mm, 64 × 64 matrix, 3.4 × 3.4 × 3.4 mm^3^ resolution, 42 axial slices).

The instructions to participants during resting state fMRI scans were identical to those received by ASD participants in the resting state MEG scans: they were told to keep their gaze on a fixation point, and remain awake. While ASD and TD groups were scanned on different magnets, all other scanning procedures and instructions to participants were identical, and subjects were scanned by the same researcher (MD).

Anatomical MRI scans for each subject were reconstructed using AFNI (Cox, 1996), and were warped to standard MNI space using FSL's nonlinear registration program *fnirt* (Andersson et al., 2007; Jenkinson et al., 2012). Standard preprocessing procedures were performed for the anatomical and resting state fMRI data, including image reconstruction, registration to MNI standard space, motion correction, spatial blurring to 6 mm full width at half maximum, spectral bandpass filtering from 0.008 to 0.08 Hz, and regression of nuisance signals derived from motion parameters, white matter, and ventricles. Time points with motion exceeding 1.5 mm from the previous time point were censored from the final analysis to reduce erroneously high correlations resulting from head motion (Power et al., 2012).

### fMRI analysis

Using the ROIs derived from the MEG source amplitude images, pairwise correlations were obtained between the BOLD time series of each possible pair of regions. These pairwise correlations were performed separately for each of the six sets of ROIs, corresponding to each of the six frequency bands for which we modeled amplitude sources. Each pairwise correlation was also compared between the ASD and TD groups using *t*-tests, to determine which nodes of each frequency-source-based network showed abnormal connectivity in ASD. Prior to these between-group tests, Pearson product moment correlation values were converted into Z-scores using the Fisher transformation.

**Figure 2:**
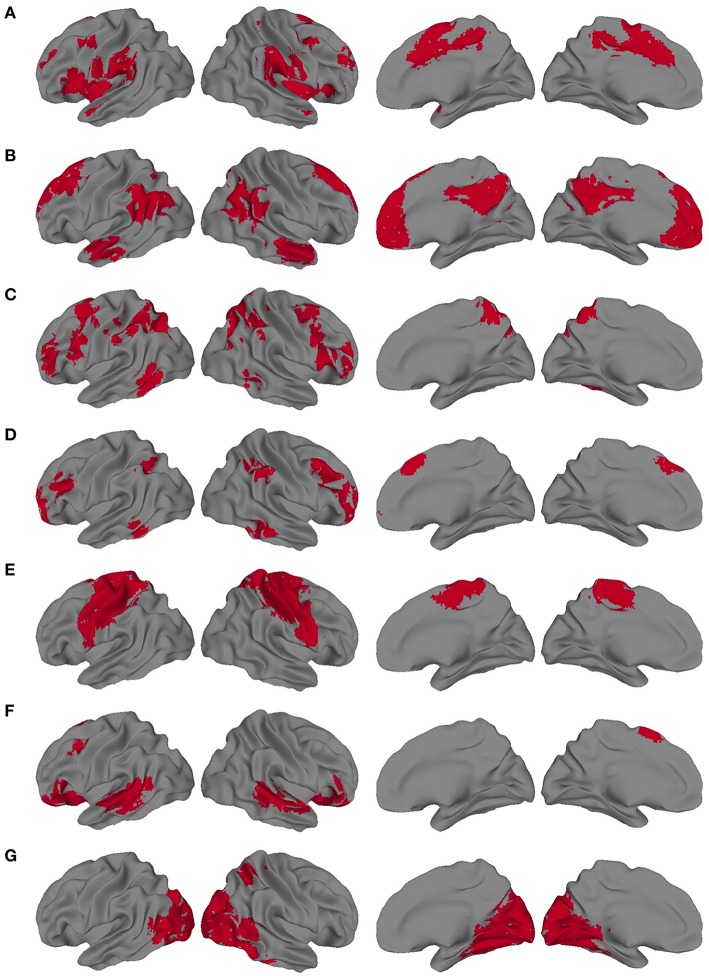
**Dilated network masks derived from the cortical parcellation from Gordon et al. (A) Cingulo-opercular, (B) Default mode network (DMN), (C) Dorsal Attention, (D) Frontoparietal, (E) Somatomotor, (F) Ventral attention, (G) Visual**.

We also sought to determine the extent to which the VESTAL-derived MEG amplitude sources overlapped with various functionally defined cortical networks. To define cortical network structure, we used a set of ROIs derived from a cortical parcellation created by Gordon et al. (2014). In that parcellation, which is shared publicly by the authors (http://www.nil.wustl.edu/labs/petersen/Resources.html), the cortex is divided into 333 separate ROIs. The authors of that paper then organized the parcels into 13 communities, which corresponded to various resting state and task-related networks and were derived from the Infomap community detection method developed by Rosvall and Bergstrom (2008). We focused on seven of the parcel communities they describe: default mode network (DMN), somatomotor (combining their separate somatomotor hand and mouth communities into one), visual, cingulo-operculum, frontoparietal, dorsal attention, and ventral attention. To adapt those regions for the present study, the parcels from each of the seven communities were combined into seven different community masks. Since the original parcels were created from a very thin layer of cortex, we then dilated our community masks by 1 voxel in all directions, using the AFNI command 3dmask_tool. The resulting community masks used for the present study are shown in Figure 2.

We conducted two follow-up correlation analyses with network-based constraints. Notably, unlike the first analysis, all between-group tests were bonferroni-corrected for multiple comparisons to a corrected *p* < 0.05. First, we looked at the pairwise correlations specifically within MEG amplitude sources that overlapped strongly with one of the seven previously discussed networks. We defined “strong” overlap as an ROI with >50% of its voxels falling within the bounds of one of our dilated network masks. For this analysis, MEG-source-based ROIs were included regardless of the frequency band from which they were derived, as it is argued that multiple frequency oscillations could emanate from the same region (Mantini et al., 2007). For instance, 1 beta source ROI, 1 gamma source ROI, and 2 high gamma source ROIs overlapped with the cingulo-opercular network, and these were examined together within one correlation matrix. ROIs included in these network-constrained correlation matrices are listed in **Table 7**.

Second, we focused on ROIs that were derived from the *same* MEG frequency band and *also* overlapped (>50% of voxels in each ROI) with the same network. Therefore, this analysis was limited to networks for which we found at least two MEG sources in the same band. Pairwise correlations were obtained for a total of nine of these sets of correlations (listed in **Table 8**). Between-group *t*-tests were performed for each pairwise correlation, and these were bonferroni-corrected for the number of multiple comparisons in each set, to a corrected *p* < 0.05.

## Results

### MEG Fast-VESTAL spectral current sources

We performed one-sample *t*-tests across all ASD subjects for the fast-VESTAL output for each frequency band. We applied a threshold to these group-level source maps in a voxel wise fashion at *p* < 0.02, and were then corrected for multiple comparisons at the cluster level to *p* < 0.01. Here we only report detailed results from the largest 10 clusters for each frequency band, although this level of correction resulted in the following number of clusters for each band: delta: 129, theta: 91, alpha: 84, beta: 128, low gamma: 19, and high gamma: 22. Anatomical labels, volumes, and MNI coordinates for the largest 10 clusters for each frequency band are listed in Tables 1–6.

**Table 1:** **Delta band ROIs: Labels, Brodmann areas (BA), peak coordinates, size (voxels), and overlaps with networks**.

**ROI label**	**No. of voxels**	**Region**	**BA**	**x**	**y**	**z**	**DMN**	**SM**	**Vis**	**FP**	**Vent. attn**.	**Dor. attn**.	**C-O**
1	134	L occipital	19	34	90	19	7 (5.2%)	0	120 (90%)	0	0	0	0
2	117	R precuneus	7	−18	78	52	0	0	51 (44%)	0	0	11 (9.4%)	0
3	106	R IPL	40	−66	39	28	44 (42%)	0	0	3 (2.8%)	0	2 (1.9%)	42 (40%)
4	83	L fusiform	37	49	69	−21	0	0	13 (16%)	5 (6%)	0	0	0
5	75	L SPL	7	28	69	61	21 (28%)	0	11 (15%)	0	0	5 (6.7%)	0
6	74	R IPL	40	−57	39	55	0	56 (76%)	0	0	0	10 (14%)	7 (9.5%)
7	73	R occipital	18	−24	99	1	0	0	61 (84%)	0	0	0	0
8	63	L cerebellum	7a	49	51	−48	0	0	0	0	0	0	0
9	60	R occipital	19	−42	84	22	13 (22%)	0	24 (40%)	0	0	0	0
10	57	L middle Frontal	9	−36	−37	46	21 (37%)	0	0	10 (18%)	0	2 (3.5%)	0

**Table 2:** **Theta band ROIs: Labels, Brodmann areas (BA), peak coordinates, size (voxels), and overlaps with networks**.

**ROI label**	**No. of voxels**	**Region**	**BA**	**x**	**y**	**z**	**DMN**	**SM**	**Vis**	**FP**	**Vent. attn**.	**Dor. attn**.	**C-O**
1	58	L occ (V4)	18	43	84	−3	0	0	51 (88%)	0	0	0	0
2	46	L orbitofrontal	11	4	−37	−27	5 (11%)	0	0	0	0	0	0
3	44	L precentral	6	58	3	37	0	44 (100%)	0	0	0	6 (14%)	12 (27%)
4	43	R angular gyrus	39	−51	69	34	30 (70%)	0	0	0	0	0	0
5	36	R postcentral	2	−57	27	52	0	33 (92%)	0	0	0	1 (2.8%)	0
6	27	L V1	17	13	96	−18	0	0	7 (26%)	0	0	0	0
7	27	R IFG	46	−57	−31	19	0	0	0	7 (26%)	12 (44%)	2 (7.4%)	0
8	26	L ITG	20	58	36	−24	0	0	12 (46%)	8 (31%)	0	0	0
9	26	R middle frontal	6	−36	−1	43	11 (42%)	0	0	0	14 (54%)	12 (46%)	8 (31%)
10	24	L temporal pole	38	55	−10	−15	13 (54%)	0	0	0	0	0	7 (29%)

**Table 3:** **Alpha band ROIs: Labels, Brodmann areas (BA), peak coordinates, size (voxels), and overlaps with networks**.

**ROI Label**	**No. of voxels**	**Region**	**BA**	**x**	**y**	**z**	**DMN**	**SM**	**Vis**	**FP**	**Vent. attn**.	**Dor. attn**.	**C-O**
1	88	L precentral	4	31	30	73	0	88 (100%)	0	0	0	8 (9.1%)	0
2	74	R Precentral	4	−42	15	52	0	72 (97%)	0	0	0	29 (39%)	6 (8.1%)
3	50	R occipital	18	−27	99	7	0	0	49 (98%)	0	0	0	0
4	42	L postcentral	2	49	33	58	0	13 (31%)	0	22 (52%)	0	0	22 (52%)
5	39	R cerebellum	8a	−12	69	−54	0	0	0	0	0	0	0
6	35	R angular gyrus	39	−54	60	34	32 (91%)	0	0	1 (2.9%)	0	1 (2.9%)	0
7	35	R precentral	6	−63	12	40	0	31 (89%)	0	0	0	0	6 (17%)
8	33	R precuneus	7	−21	78	49	0	0	19 (58%)	0	0	7 (21%)	0
9	31	L IPL	40	40	57	58	1 (3.2%)	0	4 (13%)	1 (3.2%)	0	4 (13%)	0
10	29	R precuneus	7	−3	54	67	0	5 (17%)	0	0	0	19 (66%)	0

**Table 4:** **Beta band ROIs: Labels, Brodmann areas (BA), peak coordinates, size (voxels), and overlaps with networks**.

**ROI label**	**No. of voxels**	**Region**	**BA**	**x**	**y**	**z**	**DMN**	**SM**	**Vis**	**FP**	**Vent. attn**.	**Dor. attn**.	**C-O**
1	121	R IPL	40	−45	45	46	1 (0.83%)	82 (68%)	0	0	0	68 (56%)	4 (3.3%)
2	98	R precentral	3	−45	18	55	1 (1%)	82 (84%)	0	0	1 (1%)	1 (1%)	13 (13%)
3	91	R STG	22	−60	−1	−6	13 (14%)	43 (47%)	0	0	3 (3.3%)	9 (9.9%)	29 (32%)
4	80	R IPL	40	−51	45	31	51 (64%)	1 (1.2%)	0	0	0	4 (5%)	30 (38%)
5	79	R mid frontal	9	−39	−22	34	1 (1.3%)	0	0	27 (34%)	15 (19%)	7 (8.9%)	4 (5.1%)
6	68	R SPL	7	−21	51	70	0	48 (71%)	0	0	0	39 (57%)	0
7	58	R/L cuneus	19	−6	90	28	0	0	43 (74%)	0	0	0	0
8	48	L IPL	2	43	30	43	0	46 (96%)	0	8 (17%)	0	4 (8.3%)	4 (8.3%)
9	46	L IPL	40	64	30	25	0	2 (4.3%)	0	0	0	0	46 (100%)
10	45	L pSTS	40	58	54	22	18 (40%)	0	3 (6.7%)	0	2 (4.4%)	0	3 (6.7%)

**Table 5:** **Gamma band ROIs: Labels, Brodmann areas (BA), peak coordinates, size (voxels), and overlaps with networks**.

**ROI label**	**No. of voxels**	**Region**	**BA**	**x**	**y**	**z**	**DMN**	**SM**	**Vis**	**FP**	**Vent. attn**.	**Dor. attn**.	**C-O**
1	21	R precentral	6	−63	−1	31	0	20 (95%)	0	0	0	1 (4.8%)	1 (4.8%)
2	19	L postcentral	7	4	57	67	0	0	0	0	0	8 (42%)	0
3	15	R sup temporal	42	−66	30	16	0	0	0	0	0	0	4 (27%)
4	12	R cerebellum	7a	−48	45	−33	0	0	0	0	0	0	0
5	12	L mPFC	11	13	−61	−18	9 (75%)	0	0	2 (17%)	0	0	0
6	11	R ITG	20	−63	42	−24	0	0	0	0	0	0	0
7	11	R postcentral	7	−12	54	73	0	10 (91%)	0	0	0	9 (82%)	0
8	10	L pMTG	39	−57	69	13	0	0	2 (20%)	0	0	2 (20%)	0
9	10	L STG	22	64	36	16	0	0	0	0	0	0	6 (60%)
10	9	R postcentral	43	−66	9	19	0	8 (89%)	0	0	0	0	5 (56%)

**Table 6:** **High Gamma band ROIs: Labels, Brodmann areas (BA), peak coordinates, size (voxels), and overlaps with networks**.

**ROI label**	**No. of voxels**	**Region**	**BA**	**x**	**y**	**z**	**DMN**	**SM**	**Vis**	**FP**	**Vent. attn**.	**Dor. attn**.	**C-O**
1	12	R med orbitofrontal	11	−12	−52	−24	2 (17%)	0	0	0	0	0	0
2	11	R cerebellum	8a	−33	45	−54	0	0	0	0	0	0	0
3	11	L precentral	6	25	12	73	0	9 (82%)	0	0	0	0	1 (9.1%)
4	10	L precuneus	7	4	69	64	0	0	0	0	0	1 (10%)	0
5	9	R inferior temporal	20	−63	39	−24	0	0	0	0	0	0	0
6	9	R SPL	7	−42	69	52	8 (89%)	0	0	0	0	0	0
7	9	R IPL	40	−54	39	58	0	0	0	0	0	3 (33%)	5 (56%)
8	9	L precentral	6	43	−1	58	0	5 (56%)	0	2 (22%)	0	3 (33%)	9 (100%)
9	9	L postcentral	1	49	27	61	0	9 (100%)	0	0	0	0	0
10	9	R postcentral	2	−42	36	67	0	9 (100%)	0	0	0	0	0

### FMRI functional connectivity among regions derived from MEG amplitude sources

**Figure 3:**
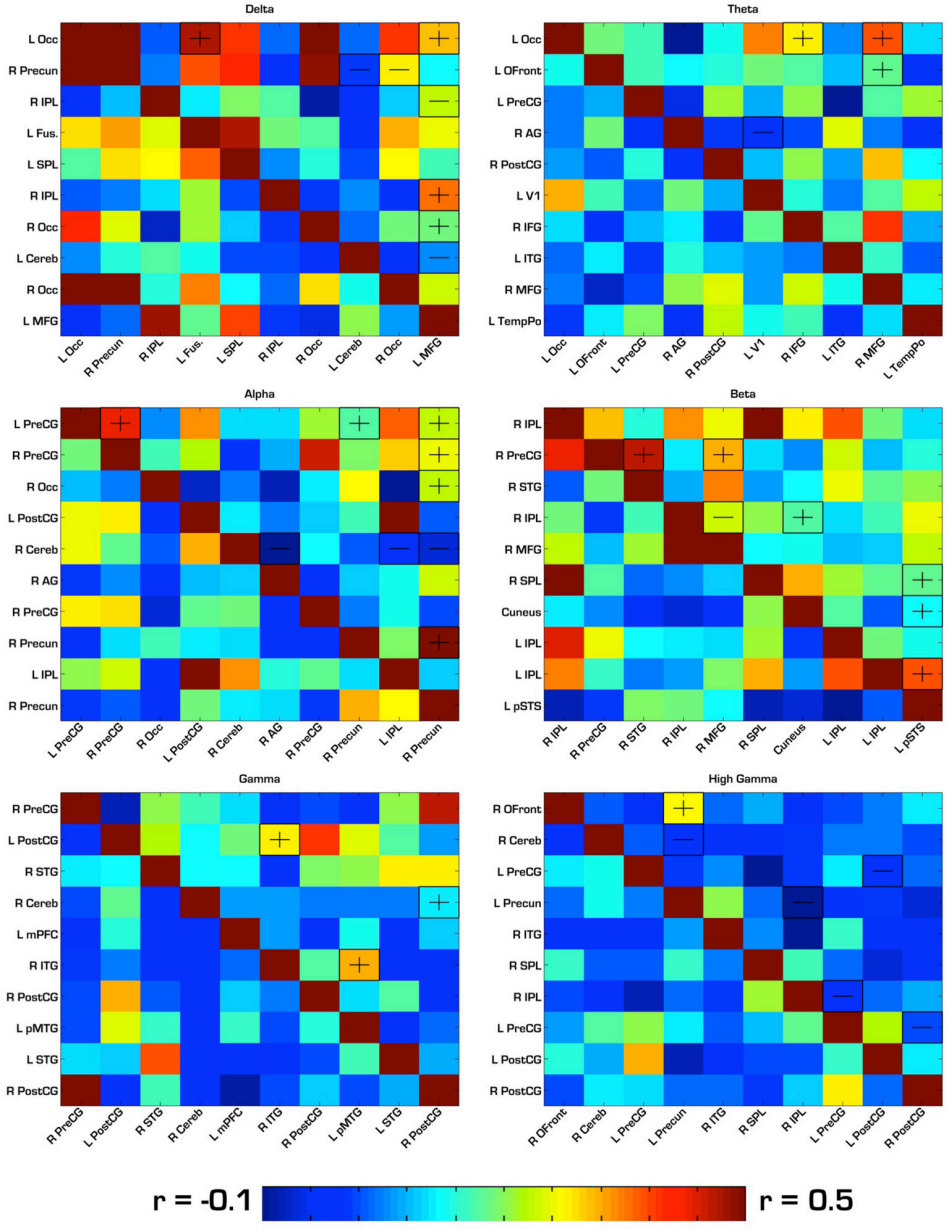
**Correlation matrices for ROIs derived from each frequency band**. ASD correlations are in the top right triangle of each matrix, while TD correlations are in the bottom left. “−” and “+” indicates ROI pairs for which ASD trended toward hypo- or hyperconnectivity, respectively. In this first analysis, none of the group differences were significant after strict Bonferroni correction. Therefore, the group differences shown here had an uncorrected *p* < 0.05. Occ, Occipital; IPL, Inferior Parietal Lobule; SPL, Superior Parietal Lobule; PreCG, Precentral Gyrus; PostCG, Postcentral Gyrus; MFG, Middle Frontal Gyrus; Cereb, Cerebellum; Precun, Precuneus; STG, Superior Temporal Gyrus; MTG, Middle Temporal Gyrus; ITG, Inferior Temporal Gyrus; AG, Angular Gyrus; Ofront, Orbito Frontal Cortex; TempPo, Temporal Pole.

Our first analysis compared the pairwise correlations of the BOLD signal among MEG-based ROIs in each frequency band of interest. For each of the six frequency bands, there were 10 ROIs, resulting in a 10 × 10 pairwise correlation matrix and a total of 45 between-group comparisons for each band. In this analysis, there were no significant between-group differences after strict bonferroni correction for multiple comparisons in each matrix (45), so we report results that passed an uncorrected *p* < 0.05. See Tables 1–6 for reference of the anatomical locations of the numbered ROIs to which we refer in each frequency band. In the delta band, ASD showed hyperconnectivity between ROIs 1–4, 1–10, 6–10, and 7–10. Also in delta, ASD showed hypoconnectivity between ROIs 2–8, 2–9, 3–10, and 8–10. In theta, ASD showed hyperconnectivity between ROIs 1–7, 1–9, and 2–9, and hypoconnectivity between ROIs 4–6. In alpha, ASD showed hyperconnectivity between ROIs 1–2, 1–8, 1–10, 2–10, 3–10, and 8–10, and hypoconnectivity between ROIs 5–6, 5–9, and 5–10. In beta, ASD showed hyperconnectivity between ROIs 2–6, 4–10, and 6–8. In gamma, ASD showed hyperconnectivity between ROIs 2–3, 2–5, 4–7, 6–10, 7–10, and 9–10, and hypoconnectivity between ROIs 4–5. In high gamma, ASD showed hyperconnectivity between ROIs 1–4, and hypoconnectivity between ROIs 2–4, 2–9, 4–7, 7–8, and 8–10. See Tables 1–6 and Figure 3 for detailed summaries of these results.

The VESTAL-derived MEG amplitude sources overlapped with several of our dilated adaptations of the Gordon parcel communities. Details about the extent of these overlaps can be found in Tables 1–6. The 10 largest delta source clusters, totaling 842 voxels, overlapped with the following networks (each listed with number of overlapping voxels): DMN: 106, somatomotor: 80, visual: 280, frontoparietal: 18, ventral attention: 0, dorsal attention: 30, cingulo-operculum: 49. The 10 largest theta source clusters, totaling 357 voxels, overlapped with the following networks (each listed with number of overlapping voxels): DMN: 59, somatomotor: 93, visual: 70, frontoparietal: 15, ventral attention: 26, dorsal attention: 21, cingulo-operculum: 27. The largest alpha source clusters, totaling 456 voxels, overlapped with the following networks (each listed with number of overlapping voxels): DMN (33), somatomotor (224), visual (72), frontoparietal (24), dorsal attention (68), and cingulo-operculum (34). The 10 largest beta source clusters, totaling 734 voxels, overlapped with the following networks (each listed with number of overlapping voxels): DMN: 85, somatomotor: 331, visual: 46, frontoparietal: 35, ventral attention: 21, dorsal attention: 132, cingulo-operculum: 133. The 10 largest gamma source clusters, totaling 130 voxels, overlapped with the following networks (each listed with number of overlapping voxels): DMN: 9, somatomotor: 38, visual: 2, frontoparietal: 2, ventral attention: 0, dorsal attention: 20, cingulo-operculum: 16. The 10 largest high gamma source clusters, totaling 98 voxels, overlapped with the following networks (each listed with number of overlapping voxels): DMN: 10, somatomotor: 32, visual: 0, frontoparietal: 2, ventral attention: 0, dorsal attention: 7, cingulo-operculum: 15.

**Figure 4:**
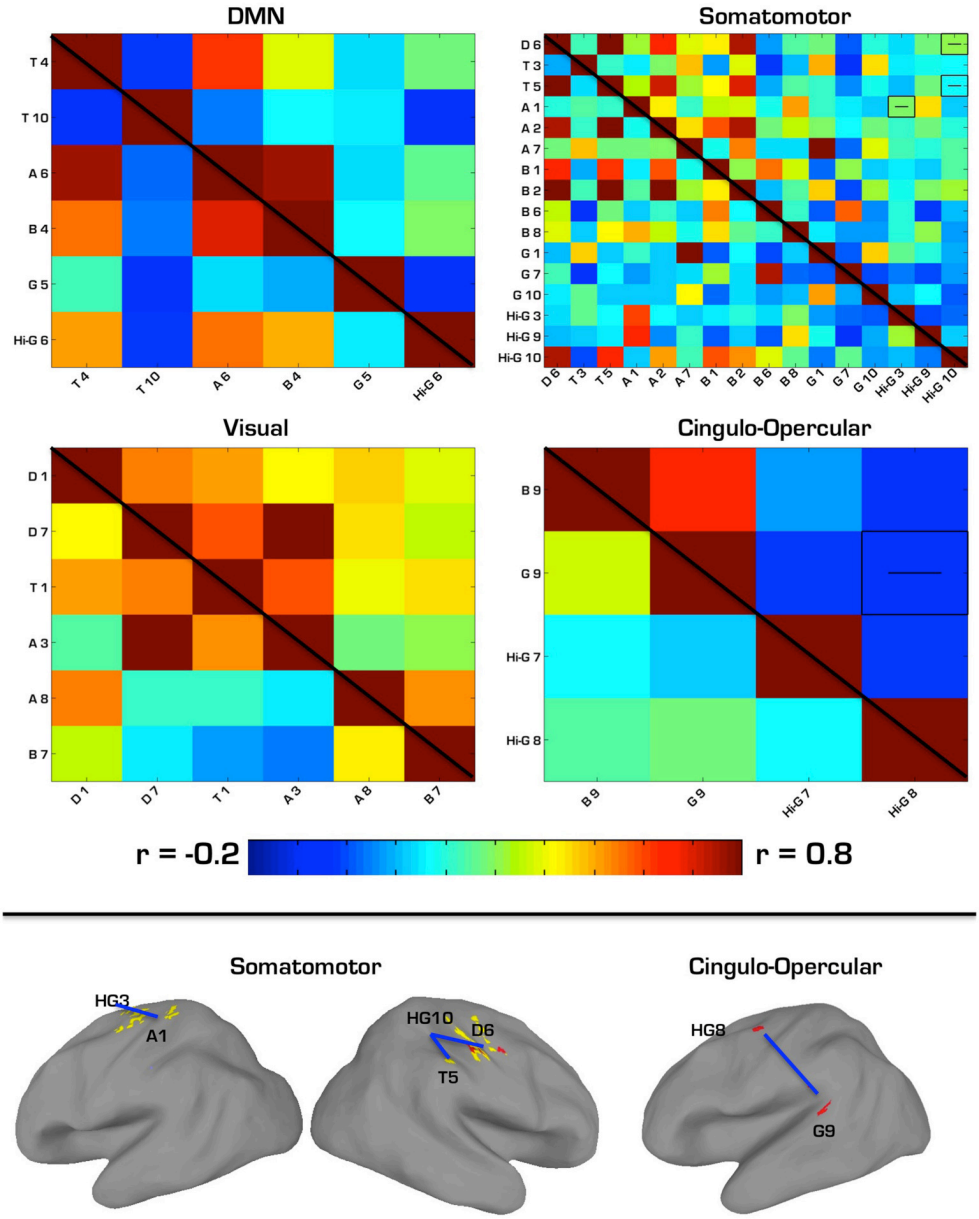
**(Top) Correlation matrices for network-constrained MEG source ROIs**. ASD correlations are in the top right triangle of each matrix, while TD correlations are in the bottom left. Pairwise correlations with significant group differences (*p* < 0.05, Bonferroni corrected) are depicted by a “−” or “+” to indicate ASD hypo- or hyperconnectivity. Labels for each ROI correspond to the first letter of each ROI's frequency band, and the number it is associated with in Tables 1–6. **(Bottom)** Regions showing significant hypoconnectivity in ASD. Regions connected by blue lines correspond to those shown in the above correlation matrices as being hyperconnected.

The first follow-up analysis included correlation matrices containing all ROIs overlapping with a specific network regardless of the MEG frequency band on which they were based. Overall, four networks overlapped with at least two ROIs each. Among the six ROIs that overlapped with the DMN (2 theta, 1 alpha, 1 beta, 1 gamma, 1 high gamma), there were no significant differences in connectivity between ASD and TD groups. Among the 16 ROIs that overlapped with the Somatomotor network (1 delta, 2 theta, 3 alpha, 4 beta, 3 gamma, 3 high gamma), there were three ROI pairs in which ASD showed *hypoconnectivity*: a delta to a high gamma ROI, theta to high gamma, and alpha to high gamma. Among the six ROIs that overlapped with the Visual network (2 delta, 1 theta, 2 alpha, 1 beta), there were no significant differences in connectivity between ASD and TD groups. Among the four ROIs that overlapped with the cingulo-operculum network (1 beta, 1 gamma, 2 high gamma), the ASD group showed *hypoconnectivity* between the gamma and a high gamma ROI. These results are further summarized in Table 7 and Figure 4.

**Table 7:** **ROIs used in network-constrained correlation matrices**.

**Network**	**Derived from MEG band**	**ROI label**	**Location**	**Percent overlap of ROI with network (%)**
DMN	Theta	4	R angular gyrus	70
	Theta	10	L temporal pole	54
	Alpha	6	R angular gyrus	91
	Beta	4	R IPL	64
	Gamma	5	L mPFC	75
	High gamma	6	R SPL	89
Somatomotor	Delta	6	R IPL	76
	Theta	3	L precentral	100
	Theta	5	R postcentral	92
	Alpha	1	L precentral	100
	Alpha	2	R Precentral	97
	Alpha	7	R precentral	89
	Beta	1	R IPL	68
	Beta	2	R precentral	84
	Beta	6	R SPL	71
	Beta	8	L IPL	96
	Gamma	1	R precentral	95
	Gamma	7	R postcentral	91
	Gamma	10	R postcentral	89
	High gamma	3	L precentral	82
	High gamma	9	L postcentral	100
	High gamma	10	R postcentral	100
Visual	Delta	1	L occipital	90
	Delta	7	R occipital	84
	Theta	1	L occ (V4)	88
	Alpha	3	R occipital	98
	Alpha	8	R precuneus	58
	Beta	7	R/L cuneus	74
Cingulo-opercular	Beta	9	L IPL	100
	Gamma	9	L STG	60
	High gamma	7	R IPL	56
	High gamma	8	L precentral	100

The second follow-up analysis looked only at correlations between sources that (a) were derived from the same MEG frequency band and (b) overlapped with the same network. Overall, nine separate sets of correlations were performed based on this criteria. Three significant effects emerged from this analysis, all surviving bonferroni-correction for multiple comparisons at *p* < 0.05. In the ASD group, two alpha ROIs were *hyperconnected* to each other within the somatomotor network, two high gamma ROIs were *hypoconnected* to each other within the somatomotor network, and two high gamma ROIs were *hypoconnected* to each other within the cingulo-operculum network. See Table 8 for a more detailed summary of results from this analysis.

**Table 8:** **ROIs used in correlation matrices constrained by both network membership and frequency band of origin**.

**Network**	**Band**	**ROI label**	**Location**	**Percent overlap of ROI with network (%)**	**ASD vs. TD *P*-values for pairwise *r***
Visual	Delta	1	L occipital	90	0.14
		7	R occipital	84	
	Alpha	3	R occipital	98	0.4
		8	R precuneus	58	
DMN	Theta	4	R angular gyrus	70	0.82
		10	L temporal pole	54	
Somatomotor	Theta	3	L precentral	100	0.52
		5	R postcentral	92	
	Alpha	1	L precentral	100	0.01 (ROIs 1 and 2)
		2	R precentral	97	0.55 (ROIs 1 and 7)
		7	R precentral	89	0.10 (ROIs 2 and 7)
	Beta	1	R IPL	68	0.02 (1 and 2), 0.83 (1 and 6), 0.47 (1 and 8)
		2	R precentral	84	0.53 (2 and 6), 0.99 (2 and 8)
		6	R SPL	71	0.97 (6 and 8)
		8	L IPL	96	
	Gamma	1	R precentral	95	0.91 (1 and 7), 0.61 (1 and 10)
		7	R postcentral	91	0.25 (7 and 10)
		10	R postcentral	89	
	High gamma	3	L precentral	82	0.001 (3 and 9)
		9	L postcentral	100	0.43 (3 and 10)
		10	R postcentral	100	0.52 (9 and 10)
Cingulo-opercular	High gamma	7	R IPL	56	0.013
		8	L precentral	100	

## Discussion

The present study used a multimodal approach to investigate brain network dynamics in children with ASD, and provides new evidence for abnormal functional connectivity in cortical networks. The integration of both MEG and fMRI data collected during a resting state enabled groupwise comparisons of functional connectivity between MEG current sources for delta, theta, alpha, beta, and gamma bands. Our primary connectivity analysis was performed on fMRI data, while the ROIs derived from MEG amplitude-sources provided a novel framework within which to test that fMRI data. One advantage of this method is that MEG allowed us to localize important hubs of fast neuronal oscillations not normally detected with fMRI. Most studies define ROIs for connectivity analyses using data from task activation studies, anatomical atlases, meta-analyses, and data driven approaches such as independent component analyses. However, no study has explored the possibility of detecting novel differences between ASD and TD samples among regions that function as neuronal oscillatory generators. Another advantage is that the use of fMRI connectivity as an outcome measure allows us to compare our results to a large body of previous evidence. Unlike MEG, the literature on fcMRI in ASD is quite extensive.

Using a spectral MEG current source-modeling algorithm (fast-VESTAL) in resting state MEG scans, we found that the spatial distribution of spectral current sources in a group of ASD participants followed general patterns reported in a previous investigation of VESTAL-derived MEG sources in healthy adults (Huang et al., 2014). Alpha sources were highly concentrated in visual and somatosensory areas, in line with previous findings on both the mu rhythm and occipital alpha (Pfurtscheller et al., 2006; Bernier et al., 2007). Beta sources overlapped with some somatosensory alpha sources, but were concentrated in more anterior and fewer posterior regions compared to alpha. Low and high gamma sources showed a wider spatial distribution, including regions such as medial prefrontal cortex, precuneus, and inferior temporal gyrus.

To determine how the distribution of spectral MEG current sources compared to previously described resting state network structure, we looked at the number of voxels from each ROI that overlapped with an existing map of cortical parcel communities from Gordon et al. (2014). Delta ROIs overlapped largely with DMN and visual areas; theta ROIs overlapped somewhat evenly with DMN, visual, and somatomotor areas; alpha ROIs overlapped largely with somatomotor and visual areas; beta ROIs overlapped mostly with somatomotor areas, but also had considerable overlap with DMN, dorsal attention, and cingulo-opercular networks; gamma ROIs overlapped mostly with somatomotor, dorsal attention, and cingulo-opercular networks; and high gamma ROIs overlapped with somatomotor and cingulo-opercular networks. The pattern of activity that emerged was one where current sources of lower frequency bands (delta, theta) were found in areas associated with resting state activity, in line with electrophysiological findings on the DMN (Mantini et al., 2007). As expected, lower frequency sources were also found in primary sensory (visual and somatomotor) cortices (Pfurtscheller et al., 2006; Bernier et al., 2007). In contrast, higher frequency bands (beta, gamma, and high gamma) were associated with areas involved in higher-order sensory integration and attention regulation such as dorsal attention and cingulo-opercular networks. Notably, these sources were derived exclusively from an ASD group, yet they displayed a general distribution consistent with previous findings in healthy adults (Huang et al., 2014). This leaves open the possibility that, while MEG current sources have a typical distribution in ASD, there exist abnormalities in their functional, coordinated activity.

The results of our first analysis of functional connectivity in resting state fMRI data using seed ROIs based on MEG amplitude sources found pairs of regions showing trends toward differences between ASD and TD groups, although none proved statistically significant after correction for multiple comparisons. There was a trend toward hyperconnectivity among alpha sources located in sensorimotor cortex, while an alpha source in the cerebellum showed a trend of hypoconnectivity to multiple regions in the parietal and temporal lobes. While not statistically significant in the present study, this trend is consistent with recent findings of cerebellum hypoconnectivity to supramodal cortical regions, including parietal and temporal regions (Khan et al., 2015).

We then performed two sets of follow-up correlation analyses based on the overlaps of our VESTAL-derived ROIs with previously described network structure. First, we looked at pairwise correlations between MEG-derived sources that overlapped with one of seven networks, based on the parcel communities described by Gordon et al. (2014). Unlike the first analysis, we used ROIs across any of the six frequency bands, as long as they overlapped with a network. This was done to highlight possible group differences in neural information encoding across multiple frequencies, in light of previous findings of abnormal coupling between different frequency bands in various disease states Voytek and Knight (2015). Hyperconnectivity of the BOLD signal between the MEG sources we identified could indicate problematic overcoupling between neuronal oscillations of different frequencies. Also unlike the first analysis, we found several between-group results that survived bonferroni correction for the number of pairwise correlations in each network's matrix. Within the somatomotor network, the ASD group showed *hypoconnectivity* in three ROIs based on high gamma MEG sources, including between a high gamma source in left postcentral gyrus to a delta source in right postcentral gyrus and to a theta source in right postcentral gyrus. Additionally, ASD was *hypoconnected* from a high gamma source in left precentral gyrus to an alpha source located more ventrally in left precentral gyrus. Within the cingulo-opercular network, the ASD group showed *hypoconnectivity* between a gamma ROI in right lateral visual cortex (V5) and a high gamma ROI in left precentral gyrus (Brodmann Area 6). In another follow-up fMRI correlation analysis, we further narrowed our focus to include only ROIs that overlapped with the same network and which were derived from the same MEG frequency band. ASD showed significant hypoconnectivity between two ROIs based on high-gamma sources within the somatomotor network. That group also showed hypoconnectivity between two other gamma ROIs (one low gamma and the other high gamma), which were instead located in the cingulo-operculum network. Thus, across both secondary analyses, there was a clear pattern of hypoconnectivity among many sources of gamma activity that overlapped with several networks. This pattern could represent the increased neural noise that is a consequence of dysfunctional inhibitory processing of GABAergic interneurons (Brock et al., 2002; Casanova et al., 2002; Brown et al., 2005; Wilson et al., 2007; Sun et al., 2012; Peiker et al., 2015). These stronger findings of within-network connectivity abnormalities support the notion of reduced within-network integration (Fair et al., 2009; Rudie et al., 2011, 2013; Fishman et al., 2014; Nebel et al., 2014), and recapitulate MEG and EEG findings on abnormal gamma connectivity, in ASD.

There were no significant differences between groups in any pairwise correlations among ROIs overlapping with the DMN, suggesting that electrophysiological activity may be relatively normal in ASD in this network. This is in contrast to some previous studies that have shown abnormal functional connectivity in DMN (Cherkassky et al., 2006; Monk et al., 2009; Lynch et al., 2013), but it could be the case that our VESTAL-derived MEG sources were located in unaffected sub regions of this network.

There are several limitations that call for caution in the interpretation of our results. First, we only collected MEG data from ASD subjects. It is possible that we would see a qualitatively different distribution of MEG amplitude sources if the VESTAL algorithms were performed on TD participants alone, or on a combination of ASD and TD participants. However, many other studies, including the initial report of the VESTAL source localization technique (Huang et al., 2014), have found source distribution patterns in neurotypical individuals were similar to those displayed by our ASD sample. Thus, while the exact locations of some current sources may differ between groups, their overall similarity to previously observed typical patterns justifies the use of these sources as the basis for the rest of our analyses. Another caveat to these results is that the TD and ASD groups were scanned on magnets of differing strength: 3T and 1.5T, respectively. Although the acquisition parameters differed between these scanners, the larger voxel size, longer TR, longer total scan length for the 1.5T scans ensured that the signal-to-noise ratio was comparable between both groups. Finally, groups were not matched for IQ, with ASD showing a lower full score on the WASI. In a follow-up analysis, we used participant IQ as a covariate in the between-group *t*-tests of pairwise functional connectivity between sources. Of the eight source pairs showing significant group differences before accounting for IQ, two pairs still showed significant differences after doing so. These included an alpha to a high gamma source within the somatomotor network (*p* = 0.019, hypoconnected in ASD), and a theta to a beta source within the visual network (*p* = 0.005, hyperconnected in ASD). The persistence of these effects after controlling for IQ is evidence that, despite a low sample size and suboptimal group matching, this novel approach to integrating resting state MEG and fMRI data was still able to detect group differences in brain connectivity between ASD and TD individuals. Thus, this approach could inform future studies with larger, well-matched samples.

The present study demonstrates a novel approach to a multimodal investigation of brain connectivity in ASD. By using an MEG-amplitude source modeling technique as the basis for fcMRI analyses, we found further evidence supporting the notion of aberrant functional connectivity in ASD. This is also is the first study to report ASD hypoconnectivity of the BOLD signal specifically between sources of MEG gamma oscillations, providing supporting evidence that dysfunctional inhibitory processing mediated by gamma oscillations may play an important role in the neuroetiology of ASD.

## Author contributions

MD collected fMRI and MEG data, preprocessed and analyzed data, and wrote much of the manuscript. RG collected MEG data, preprocessed MEG data, and contributed to the manuscript. MH contributed the VESTAL algorithm that was used to analyze MEG data, and trained other authors in the use of VESTAL. JP collected MEG data, advised during data preprocessing and analysis steps, and contributed to the manuscript.

### Conflict of interest statement

The authors declare that the research was conducted in the absence of any commercial or financial relationships that could be construed as a potential conflict of interest.
